# O-GlcNAcylation is essential for therapeutic mitochondrial transplantation

**DOI:** 10.1038/s43856-023-00402-w

**Published:** 2023-11-25

**Authors:** Ji Hyun Park, Masayoshi Tanaka, Takafumi Nakano, Ester Licastro, Yoshihiko Nakamura, Wenlu Li, Elga Esposito, Emiri T. Mandeville, Sherry Hsiang-Yi Chou, MingMing Ning, Eng H. Lo, Kazuhide Hayakawa

**Affiliations:** 1 Neuroprotection Research Laboratory, Departments of Radiology and Neurology, Massachusetts General Hospital and Harvard Medical School, Charlestown, MA USA; 2 Departments of Critical Care Medicine, Neurology and Neurosurgery, University of Pittsburgh, Pittsburgh, PA USA; 3 Department of Neurology, Brigham and Women’s Hospital and Harvard Medical School, Boston, MA USA; 4 Clinical Proteomics Research Center, Department of Neurology, Massachusetts General Hospital and Harvard Medical School, Boston, MA USA

**Keywords:** Mitochondria, Post-translational modifications, Stroke

## Abstract

**Background:**

Transplantation of mitochondria is increasingly explored as a novel therapy in central nervous system (CNS) injury and disease. However, there are limitations in safety and efficacy because mitochondria are vulnerable in extracellular environments and damaged mitochondria can induce unfavorable danger signals.

**Methods:**

Mitochondrial O-GlcNAc-modification was amplified by recombinant O-GlcNAc transferase (OGT) and UDP-GlcNAc. O-GlcNAcylated mitochondrial proteins were identified by mass spectrometry and the antiglycation ability of O-GlcNAcylated DJ1 was determined by loss-of-function via mutagenesis. Therapeutic efficacy of O-GlcNAcylated mitochondria was assessed in a mouse model of transient focal cerebral ischemia-reperfusion. To explore translational potential, we evaluated O-GlcNAcylated DJ1 in CSF collected from patients with subarachnoid hemorrhagic stroke (SAH).

**Results:**

We show that isolated mitochondria are susceptible to advanced glycation end product (AGE) modification, and these glycated mitochondria induce the receptor for advanced glycation end product (RAGE)-mediated autophagy and oxidative stress when transferred into neurons. However, modifying mitochondria with O-GlcNAcylation counteracts glycation, diminishes RAGE-mediated effects, and improves viability of mitochondria recipient neurons. In a mouse model of stroke, treatment with extracellular mitochondria modified by O-GlcNAcylation reduces neuronal injury and improves neurologic deficits. In cerebrospinal fluid (CSF) samples from SAH patients, levels of O-GlcNAcylation in extracellular mitochondria correlate with better clinical outcomes.

**Conclusions:**

These findings suggest that AGE-modification in extracellular mitochondria may induce danger signals, but O-GlcNAcylation can prevent glycation and improve the therapeutic efficacy of transplanted mitochondria in the CNS.

## Plain language summary

Mitochondria are the part of a cell that generate most of its energy to perform its functions. In injury or disease, mitochondrial function can become disrupted. Transplantation of healthy mitochondria is being explored as a potential therapy to replace damaged mitochondria and restore normal cellular function. However, this approach is difficult to perform because mitochondria are not able to maintain their healthy state outside of cells. Here, we show that one of the reasons for this is due to a molecular process called advanced glycation end product modification. We show that simple modification of mitochondria with a sugar prevents this process and helps to improve the success of therapeutic mitochondrial transplantation in cells and in a mouse model of stroke. Our findings may help to guide future efforts to develop therapies based on mitochondrial transplantation.

## Abstract (web summary)

Park et al. assess the therapeutic efficacy of mitochondrial transplantation with or without O-GlcNAc modification of isolated mitochondria. O-GlcNAcylation can prevent glycation while improving both the functional properties of mitochondria and the neuroprotective efficacy of transplantation in a mouse model of focal cerebral ischemia.

## Introduction

Mitochondria are essential for maintaining intracellular function in high-metabolism organs such as the central nervous system (CNS)^1^. In the context of CNS injury or disease, mitochondrial dysfunction is a major contributor to inflammation and cell death^2,3^. Therefore, finding ways to restore mitochondrial integrity continues to be an important therapeutic strategy for many CNS disorders including stroke^4^, intracerebral hemorrhage^5^, spinal cord injury^6^, Alzheimer’s disease^7^, Parkinson’s disease^8^, and amyotrophic lateral sclerosis^9^.

Recently, accumulating findings in cell, animal and human studies suggest that extracellular mitochondria may play an important role for non-cell-autonomous signaling^10,11^, and the transplantation of exogenous mitochondria is being pursued as a novel therapy for CNS injury and disease^12,13^. However, caveats and limitations exist because mitochondria may be altered in the extracellular environment, and dysfunctional mitochondrial transfer can worsen neuroinflammation^14^.

Almost all CNS diseases occur in the context of aging and cardiovascular comorbidities. In this regard, advanced glycation end products (AGE) formed by irreversible non-enzymatic glycosylation is recognized to be a major biomolecule that can induce neurotoxicity^15^. Is it possible that extracellular mitochondria can also be AGE-modified, thus limiting the efficacy of mitochondrial transfer? And if so, can we find ways to overcome this hurdle?

O-GlcNAcylation is the process that attaches O-linked β-N-acetylglucosamine to serine and/or threonine side chains of the polypeptide backbone in nuclear, mitochondrial and cytoplasmic proteins^16^. The addition and removal of O-GlcNAc moieties cycles is regulated by O-GlcNAc transferase (OGT) and O-GlcNAcase and the optimum range of the modification is critical to coordinate mitochondrial energy metabolism through modulating components of TCA cycle and mitochondrial respiratory chain^17^. In this study, we used a combination of cells, mouse models, and human studies to demonstrate that modifying extracellular mitochondria with O-GlcNAcylation protected them against glycation, and improved the therapeutic efficacy of mitochondrial transplantation in the CNS.

## Methods

### Reagents

β-Nicotinamide adenine dinucleotide sodium salt (Sigma Aldrich, N0632-1G), Mitotracker Deep Red FM (Thermo Fisher Scientific, M22426).

### In vivo animal studies

All experiments were performed under approved Institutional Animal Care and Use Committee protocol (2016N000493, 2016N000163, 2010N000228) in accordance with National Institutes of Health guidelines and with the United States Public Health Service’s Policy on Human Care and Use of Laboratory Animals and following Animals in Research: Reporting In vivo Experiments (ARRIVE) guidelines. Because female mice must be tested across the estrous cycle and are more variable than males, male mice were used in this proof-of-concept study. All male C57BL/6J mice were (up to 4 mice per cage) maintained in a controlled pathogen-free/germ-free environment with a temperature of 68–73 °F, 12/12 h light/dark cycle, 30–70% humidity, and food (Prolab Isopro RMH3000 Irradiated, 3003219-249) and water provided ad libitum. Mice were anesthetized using isoflurane (2–3% induction, 1–1.5% maintenance), and received buprenorphine (0.05–0.1 mg/kg, intraperitoneal injection) pre- and post-surgery. Mice were deeply anesthetized with isoflurane (3%), and then subjected to cardiac perfusion before brain removal. Our methods also included randomization, blinding and statistical criteria consistent with ARRIVE guidelines (ARRIVE). In a mouse model of focal ischemia, male C57BL/6J mice (12–14 weeks) are anesthetized with 5% to 1% isoflurane, and rectal temperatures and cerebral blood flow are monitored. After midline skin incision, 7–0 nylon monofilament coated with silicon resin was introduced through a small incision into the common carotid artery. Cerebral ischemia was assessed by Laser Doppler flowmetry and by examining forelimb flexion after the mice recovered from anesthesia. After occluding the MCA for 60 min in mice, the monofilament suture was gently withdrawn in order to restore blood flow, and LDF values were recorded for 10 min after reperfusion. For mitochondrial transplant, mitochondria were isolated from CC in male C57BL/6J mice by Mitochondria Isolation Kit for Tissue (Thermo Fisher Scientific, 89801), followed by filtrating isolated fraction with 1.2 μm filter in order to exclude cellular debris. In order to modify mitochondria with O-GlcNAc, UDP-GlcNAc (0.5 mM, Sigma, U4375) and recombinant O-GlcNAc transferase (OGT, 0.5 μg, R&D systems, 8446-GT-010) or Thiamet G (2 μM, Tocris Bioscience, 4390) were co-incubated with isolated mitochondria for 30 min at 37 ^o^C within 10 mM HEPES (pH7.5) buffer containing 0.25 M sucrose, 1 mM ATP, 0.1 mM ADP, 5 mM sodium succinate, and 2 mM K_2_HPO_4_. Modified or unmodified mitochondria (each 6 μg/5 μL) were resuspended in PBS and stereotaxically injected into lateral ventricles after reperfusion.

### *Behavioral test:* neurological severity scores

Functional outcome after stroke was assessed by a 10-point neurological severity score (NSS). The score consists of ten individual clinical parameters, including tasks on motor function, alertness, and physiological behavior, whereby 1 point is given for failure of one task and no points are given for success. A maximal NSS of 10 points indicates severe neurological dysfunction, with failure at all tasks. *Foot-fault test*: Mice were performed this test on elevated 8 × 10 square grids of 2 cm × 2 cm size. Mice placed their forelimb and hindlimb on the wire while moving along the grid. The paw may fall or slip during moving between grid and grid and this was recorded as a foot fault. The total number of steps for 120 s was counted, and total number of foot faults for each paw was recorded.

### SAH patient recruitment and sample collection

All patients were enrolled after informed consent and in accordance with institutional review board approved IRB from Mass General Brigham (2007P002168). Consecutive patients within 96 h of onset of spontaneous, non-traumatic SAH were prospectively enrolled into a large SAH biomarker cohort study and serial blood and available CSF were collected and stored. CSF samples were collected only if patient has an external ventricular drain (EVD) placed for clinical indications. Patients with traumatic SAH, pregnancy, end-stage renal or hepatic disease, intracranial malignancies or infectious meningitis were excluded. In both patient populations, CSF samples were immediately centrifuged at room temperature at 3900 RPM for 15 min and supernatants were aliquoted and immediately frozen on dry ice and stored at −80 ^o^C until analysis. Long-term functional outcome were prospectively assessed at 3-month using modified Rankin score (mRS) via standardized telephone interview. A standardized script was used to perform mRS scoring to reduce inter-rater variability. Poor outcome was defined as mRS > 2.

### Primary neuron cultures

Primary neuron cultures were prepared from cerebral cortices of E17-day-old C57BL/6J mouse embryos. Briefly, cortices were dissected and dissociated using papain dissociation system (Worthington Biochemical Corporation, LK003150). Cells were spread on plates coated with poly-D-lysine (Sigma, P7886) and cultured in Dulbecco’s modified Eagle medium (NBM, Life Technology, 11965-084) containing 25 mM glucose, 4 mM glutamine, 1 mM sodium pyruvate, and 5% fetal bovine serum at a density of 2 × 10^5^ cells/mL (1 mL for 12 well format, 0.5 mL for 24 well format). At 24 h after seeding, the medium was changed to Neurobasal medium (Invitrogen, 21103-049) supplemented with B-27 (Invitrogen, 17504044) and 0.5 mM glutamine. Cells were cultured at 37 °C in a humidified chamber of 95% air and 5% CO_2_. Cultures were used for experiments from 7 to 10 days after seeding.

### Primary astrocyte cultures

Primary astrocyte cultures were prepared from cerebral cortices of 2-day-old neonatal Sprague-Dawley rats. Briefly, dissociated cortical cells were suspended in Dulbecco’s modified Eagle medium (DMEM, Life Technology, 11965-084) containing 25 mM glucose, 4 mM glutamine, 1 mM sodium pyruvate, and 10% fetal bovine serum and plated on uncoated 25 cm^2^ flasks at a density of 6×10^5^ cells/cm2. Monolayers of type 1 astrocytes were obtained 12–14 days after plating. Non-astrocytic cells such as microglia and neurons were detached from the flasks by shaking and removed by changing the medium. 4 mL of astrocyte conditioned media (ACM) were collected from 85–90% confluent astrocytes (approximately 6 × 10^5^ cells/cm^2^ in T25 flasks) from one T25 flask and ACM collected by five T25 flasks were concentrated by Vivaspin 20 (SARTORIUS, VS2041). Final one mL of concentrated media was assessed by flow cytometry analysis.

### HEK293 cultures

HEK293 cells were cultured in Dulbecco’s modified Eagle medium (DMEM, Life Technology, 11965-084) with 10% fetal bovine serum at 37 °C in a humidified chamber of 95% air and 5% CO_2_. One day after plating, cells were transfected with DJ1 WT or T19A construct using Lipofectamine 3000 reagent according to the manufacturer’s instructions (Thermo Fisher Scientific, L3000015). After 24 h, cells were treated with NAD for 24 h, and mitochondria were isolated.

### AGE assays

Fluorometric assay was used for mitochondrial Advanced Glycation End Products (AGEs) measurement (AGEs Assay Kit; BioVision, K929-100). Briefly, 5 µg of mitochondria isolated from DJ1 WT or T19A over-expressing cells were incubated in the 96-well plate with or without MGO (50 µM) at room temperature for 30 min. Then 100 µl of AGEs assay buffer was added to the each well and incubated at room temperature for 5 min. Florescence (Ex 360 nm/Em 460 nm) was measured using a fluorescence microplate reader. The fluorescence of the background control (BSA, 5 µg) was used as a reference.

### Oxygen-glucose deprivation (OGD) and reoxygenation

OGD experiments were performed using a specialized, humidified chamber (Heidolph, incubator 1000, Brinkmann Instruments, Westbury, NY) kept at 37 ^o^C, which contained an anaerobic gas mixture (90% N_2_, 5% H_2_, and 5% CO_2_). To initiate OGD, culture medium was replaced with deoxygenated, glucose-free Dulbecco’s modified Eagle medium (Life Technology, 11966-025). After 2 h challenge, cultures were removed from the anaerobic chamber, and the OGD solution in the cultures was replaced with maintenance medium. Cells were then allowed to recover for 18 h (for neurotoxicity assay).

### Mitochondria membrane potential measurement

To monitor mitochondrial health, JC1 dye (invitrogen, T-3168) was used to assess mitochondrial membrane potential. ACM were incubated with JC1 (0.8 μM) for 30 min at 37 °C. JC1 dye exhibits potential-dependent accumulation in mitochondria, indicated by fluorescence emission shift from green (Ex 485 nm/Em 516 nm) to red (Ex 579 nm/Em 599 nm). Mitochondria membrane potential was determined by the fluorescent ratio with a fluorescent microplate reader.

### Oxygen consumption analysis

Real time basal oxygen consumption rate in isolated mitochondria or damaged neurons were measured by Oxygen Consumption Rate Assay kit (Cayman Chemicals, 600800) according to the instruction provided by Cayman Chemicals. Briefly, Neurons (2 × 10^5^ cells/well/100 μL) or isolated mitochondria (10 μg/100 μL) were prepared in clear bottom black 96 well plates, and oxygen sensor probe (10 μL) was added into each well. After covering with 100 μL of Oil, the plate were read with filter combination of 340 nm for excitation and 642 nm of emission at 30 ^o^C. Oligomycin (1 μM) or antimycin A (1 μM) was treated during OCR measurement.

### FACS analysis

Standard FACS analysis was performed by BD Fortessa. Astrocyte-conditioned medium (ACM) was collected from rat cortical astrocytes. Briefly, ACM were centrifuged by 2000 rpm for 5 min in order to exclude cellular debris. When ACM were collected from astrocytes labeled by mitochondria GFP, samples were directly analyzed by BD Fortessa following the centrifugation. In order to detect mitochondrial DNA and mitochondrial O-GlcNAc, ACM were incubated with Mitotracker Deep Red (50 nM) and O-GlcNAc FITC antibody (2 µg/mL) for 30 min at 37 ^o^C. For CSF measurements, Mitotracker Deep Red (1 µL, 5 mM) was added in CSF sample (100 µL) for 30 min at 37 ^o^C. FACS analysis was performed using an unstained or phenotype control for determining appropriate gates, voltages, and compensations required in multivariate flow cytometry.

### Western blot analysis

Each sample was loaded onto 4–20% Tris-glycine gels. After electrophoresis and transferring to nitrocellulose membranes, the membranes were blocked in Tris-buffered saline containing 0.1% Tween 20 and 0.2% I-block (Tropix, T2015) for 90 min at room temperature. Membranes were then incubated overnight at 4 °C with following primary antibodies, anti-β-actin (1:1,000, Sigma-aldrich A5441), anti-CD38 antibody (1:500, Santacruz, sc-7049), anti-TOM40 (1:200, Santacruz, sc-11414), anti-O-GlcNAc (1:1000, Abcam, ab2739), anti-DJ1 (1:1000, Cell signaling, 5933S), anti-Atg12, anti-Atg5, and anti-LC3A/B (1:1000, Cell signaling, 4445T). After incubation with peroxidase-conjugated secondary antibodies, visualization was enhanced by chemiluminescence (GE Healthcare, NA931- anti-mouse, or NA934- anti-rabbit, or NA935- anti-rat). Optical density was assessed using the NIH Image analysis software.

### Immunocytochemistry and immunohistochemistry

After staining with primary antibody fluorescent-tagged secondary antibody, nuclei were counterstained with or without 4,6-diamidino-2-phenylindole (DAPI), and coverslips were placed. Immunostaining images were obtained with a fluorescence microscope (Nikon ECLIPSE Ti-S) or Nikon A1SiR Confocal Microscope.

### Human endocytosis array

Gene profiling was performed using GeneQueryHumn Endocytosis qPCR array kit (#GK022, ScienCell) by following the manufactural instruction. Briefly, human neurons were subjected to 2h OGD and human astrocyte-derived mitochondria with or without O-GlcNAc-modification were added onto these neurons. At 3 h post-mitochondria treatment, RNA was extracted and processed the array.

### mtDNA detection

Genomic and mtDNA were extracted by alkaline lysis/neutralization followed by isopropanol precipitation from conditioned media (Thermo Fisher Scientific, 4478359). Relative levels of mtDNA to genomic DNA were determined by amplifying MT-ND1 gene (Applied Biosystems, Rn03296764) and HBB (beta-globin) gene (Applied Biosystems, Rn04223896).

### siRNA and plasmid transfection

Control siRNA, CD38 siRNA were obtained from Santa Cruz Biotechnology, Inc. CA. USA. Control siRNA (sc-37007) consists of a scrambled sequence that will not lead to the specific degradation of any known cellular mRNA. Mouse CD38 siRNA (sc-37246) is a pool of three target-specific 19–25 nt siRNAs designed to knock down gene expression. The sequences for mouse CD38 siRNAs are designed as followed; 5’-GUGUACUACCAACAUUCAA-3’, 5’-GUGUGUCUUUAGUAGGUAU-3’, 5’-CCAGUUUGUGAUUGUUGA-3’. Five days after cerebral ischemia, mice were stereotaxically injected control siRNA or CD38 siRNA in intra-cerebro-ventricular (i.c.v.). The placement coordinates for the left lateral ventricle were anteroposterior: 0.5 mm from bregma, lateral: 0.8 mm from bregma, depth: 2.5 mm from the skull surface. siRNAs for i.c.v. injection were prepared according to the in vivo siRNA transfection protocol for brain delivery from PolyPlus Transfection (403-05). 4 μL of the siRNA complexes were i.c.v.-injected as 1 μL/min of flow rate of mice under anesthesia. For the ATP5O, SOD2 and DJ1/PARK7 plasmids transfection, Lipofectamine 3000 reagent was used according to the manufacturer’s instructions.

### Site-directed mutagenesis

ATP5O, SOD2 and DJ1/PARK7 plasmids were gifted from Dr. Roger Reeves (Addgene plasmid #53812), Dr. Bert Vogelstein (Addgene plasmid #16612) and Dr. Mark Cookson (Addgene plasmid #29347), respectively. Point mutations in ATP5O (T43A/S47A/S50A), SOD2 (T79A) and DJ1/PARK7 (T19A) were generated using the Q5 Site-Directed Mutagenesis Kit (NEB, E0554S) according to the manufacturer’s instructions and the following primers (mutated bases in lower case bold).

ATP5O (T43A/S47A/S50A)

F-**g**CAGCTCTTTAT**g**CTGCTGCA**g**CAAAACAGAAT

R-GGCATAGCGACCTTCAATACCGTATACCTGAAC

SOD2 (T79A)

F-GCCAAGGGAGATGTT**g**CAGCCCAGATAGCTCTT

R-CAACGCCTCCTGGTACTTCTCCTCGGTGACGTT

DJ1/PARK7 (T19A)

F-GCAGAGGAAATGGAG**g**CGGTCATCCCTGTAGAT

R-TCCTTTAGCCAGGATGACCAGAGCTCTTTTGGA

### Statistics and Reproducibility

Results were expressed as mean ± SD. In vitro experiments were performed in duplicate or triplicate or sample-combined depending on the assay sensitivity, repeated at least three times independently. Sample size was predetermined using the software available online: https://www.danielsoper.com/statcalc/calculator.aspx?id=47. The calculation was based on Cohen’s d value where SD and average were estimated from our historical and preliminary data. All of in vivo experiments were performed with full blinding, allocation concealment and randomization. Before treatment, 4 mice were allocated in a cage and randomly picked up for the procedure. To blind the treatment, mitochondrial administration was performed by Y.N., and sampling followed by endpoint assessments were conducted by other investigators. After obtaining all data, Y.N. disclosed experimental groups. All animals were included in this study. GraphPad Prism version 9 was used overall statistical analysis in this study. When only two groups were compared, unpaired t-test (two-tailed) was used. Multiple comparisons were evaluated by one-way or two-way ANOVA followed by Tukey’s test or Sidak test. *P* < 0.05 was considered to be statistically significant.

### Reporting summary

Further information on research design is available in the Nature Portfolio Reporting Summary linked to this article.

## Results

### O-GlcNAc modification promotes viable mitochondria transfer via preventing glycation

**Fig. 1:**
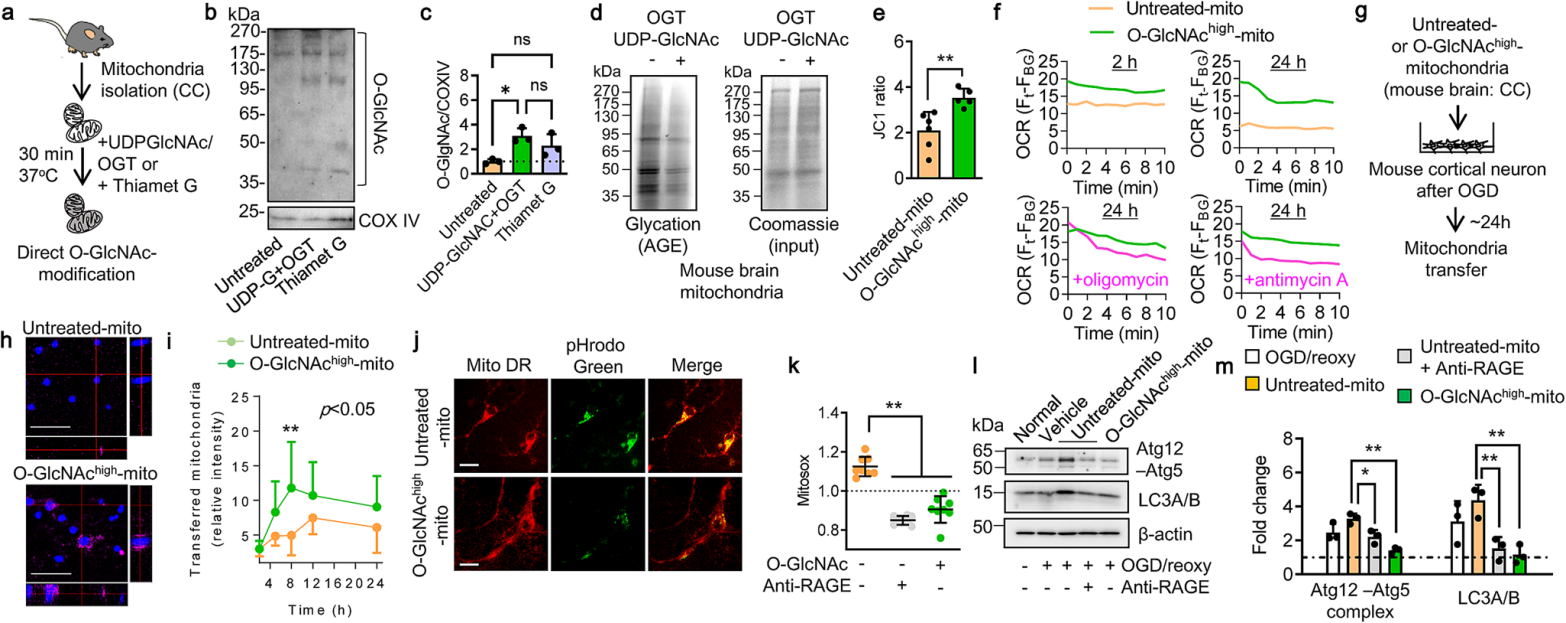
O-GlcNAcylation prevents mitochondrial protein glycation and promotes viable mitochondria transfer. **a** Mitochondria isolated from intact cerebral cortex in male C57BL/6J mice were incubated with UDP-GlcNAc (32.5 µg) and O-GlcNAc transferase (OGT) (0.66 μg) or Thiamet G (2 μM) for 30 min at 37 ^o^C. CC: cerebral cortex of male C57BL/6J. **b** Western blot confirmed that O-GlcNAc-modification of DJ1 was amplified by the treatments with OGT and UDP-GlcNAc or Thiamet G in isolated mitochondria. Note: Loading controls (COX IV) were run on separate membranes. **c** Quantification of O-GlcNAcylated protein showed that O-GlcNAcylation was amplified by treatment with UDP-GlcNAc/OGT or Thiamet G compared to untreated control mitochondria (*n* = 3). All values are mean ± SD. One-way ANOVA: F(2,6) = 7.795, *P* = 0.0215 followed by Tukey test, *P < 0.05. **d** Untreated control mitochondria exacerbated protein glycation compared to O-GlcNAcylated mitochondria. There was no difference in total mitochondrial protein loaded in the gel (input). Note: Input was run on separate membrane. **e** Mitochondrial membrane potential was assessed by JC1. O-GlcNAcylated mitochondria (O-GlcNAc^high^-mito, *n* = 5) had higher JC1 values compared to untreated control mitochondria (Untreated-mito, *n* = 6). All values are mean ± SD. Unpaired *t* test (two-tails), **P *=* 0.0059. **f** Mitochondria treated with OGT and UDP-GlcNAc showed higher basal level of oxygen consumption activity compared to untreated mitochondria at 2 h after isolation. Notably, O-GlcNAc-modified mitochondria maintain their basal level of oxygen consumption for 24 h, whereas untreated mitochondria diminished the activity. Olygomycin (1 μM) or antimycin A (1 μM) decreased the respiratory activity in O-GlcNAcylated mitochondria at 24 h. Note: mitochondria were suspended in 10 mM HEPES (pH7.5) buffer containing 0.25 M sucrose, 1 mM ATP, 0.1 mM ADP, 5 mM sodium succinate, and 2 mM K_2_HPO_4_ and stored at 4 °C until the assay. **g** O-GlcNAc-modified mitochondria (O-GlcNAc^high^-mitochondria, 2 µg/well) or unmodified control mitochondria susceptible to glycation (Untreated mitochondria, 2 µg/well) were treated in mouse cortical neurons following oxygen-glucose deprivation (OGD) for 2 h. Note: UDP-GlcNAc and OGT were removed by washing mitochondria twice by PBS before adding mitochondria onto neurons. **h** Confocal microscopy analysis confirmed that untreated or GlcNAc^high^-mitochondria were found within neurons at 16 h post-mitochondria treatment. Scale: 100 µm. **i** O-GlcNAc^high^-mitochondria were more remained in the recipient neurons compared to untreated control mitochondria (*n* = 7). All values are mean ± SD. Two-way ANOVA (time course: *P* < 0.0001, untreated vs O-GlcNAc^high^: *P* = 0.0166) followed by Sidak test, **P < 0.01. **j** Untreated control mitochondria induced robust green fluorescence reflecting acidic lysosomes, while O-GlcNAc^high^-mitochondria did not show detectable signals. Scale: 100 µm. **k** Untreated control mitochondria increased mitochondrial superoxide in the recipient neurons via receptor for advanced glycation end products (RAGE) at 8 h after transfer. But O-GlcNAc-mitochondria did not induce mitochondrial superoxide in the treated neurons (*n* = 8). All values are mean ± SD. One-way ANOVA: F(2,21) = 65.81, *P* < 0.0001 followed by Tukey test, **P<0.01. **l** Western blot analysis to assess autophagy markers (Atg12–5 complex, LC3A/B). Note: β-actin signals were obtained by reuse of the membranes after stripping Atg12-Atg5 signals. **m** Quantification data showed that untreated control mitochondria increased autophagy markers (Atg12–5 complex, LC3A/B) via RAGE at 8 h after transfer. But O-GlcNAc-mitochondria did not induce autophagy responses in the treated neurons (*n* = 3). All values are mean ± SD. Atg12-Atg5: One-way ANOVA: F(3,8) = 12.63, *P* = 0.0021 followed by Tukey test, *P < 0.05, **P < 0.01. LC3A/B: One-way ANOVA: *F*(3,8) = 8.548, *P* = 0.0071 followed by Tukey test, **P < 0.01.

Mitochondria were isolated from intact cerebral cortex (CC) of male C57BL/6J mice and suspended in 10 mM HEPES (pH7.5) buffer containing 0.25 M sucrose, 1 mM ATP, 0.1 mM ADP, 5 mM sodium succinate, and 2 mM K_2_HPO_4_ (Fig. 1a). Western blot analysis revealed that treatment with recombinant OGT and UDP-GlcNAc or Thiamet G increased O-GlcNAc-modification at approximately 3-fold or 2.5-fold in the isolated mitochondria compared with untreated mitochondria (Fig. 1b, c, Supplementary Fig. 1a). Next, we evaluated mitochondrial protein glycation. Western blots showed significant glycation of numerous protein bands in untreated mitochondria (Fig. 1d). However, when extracellular mitochondria were treated with OGT and UDP-GlcNAc to increase O-GlcNAcylation, the levels of AGE-modification in mitochondrial protein were decreased (Fig. 1d, Supplementary Fig. 1b). Concomitantly, untreated control mitochondria showed lower and variable mitochondrial membrane potential, but O-GlcNAcylation in mitochondria (O-GlcNAc^high^-mitochondria) improved membrane potential (Fig. 1e). Moreover, mitochondria treated with OGT and UDP-GlcNAc showed higher basal level of oxygen consumption activity compared to untreated mitochondria. Notably, O-GlcNAc-modified mitochondria maintained their basal level of oxygen consumption for 24 h, whereas untreated control mitochondria decreased the activity (Fig. 1f). Importantly, the mitochondrial respiratory activity at 24 h was decreased by inhibiting F1Fo ATP synthase with oligomycin or by blocking electron transfer at complex III with antimycin A in O-GlcNAc-modified mitochondria (Fig. 1f), implicating that O-GlcNAc modification may partly preserve mitochondrial respiratory chain complexes.

Next, we asked whether preventing AGE-modification with O-GlcNAcylation can improve the therapeutic efficacy of mitochondrial transfer in the CNS. Mouse primary neurons were damaged with oxygen-glucose deprivation (OGD) for 2 h, then untreated control mitochondria or O-GlcNAc^high^-mitochondria were added onto the injured neurons (Fig. 1g). It is important to note that substrates that were used for O-GlcNAcylation in isolated mitochondria were removed by washing mitochondria twice by PBS before adding onto neurons. Confocal microscopy analysis demonstrated that untreated or O-GlcNAc^high^-mitochondria were found within damaged neurons subjected to 2 h OGD (Fig. 1h). Consistently, the fluorescent intensity measurements demonstrated that O-GlcNAc^high^-mitochondria showed better accumulation and retention compared with untreated mitochondria (Fig. 1i). Furthermore, untreated mitochondria appeared to induce the formation of lysosomes in the recipient neurons (Fig. 1j). Mitosox and western blot analysis demonstrated that untreated mitochondria increased mitochondrial reactive oxygen species (Fig. 1k) and autophagy markers such as Atg12-Atg5 complex and LC3A/B (Fig. 1l, m, Supplementary Fig. 1c). Blockade of neuronal receptor for advanced glycation end products (RAGE) diminished these effects (Fig. 1l, m, Supplementary Fig. 1c). In contrast, O-GlcNAc^high^-mitochondria did not increase the mitochondrial stress response and autophagic degradation in the recipient neurons (Fig. 1l, m, Supplementary Fig. 1c). Taken together, these data suggest that extracellular mitochondria may be susceptible to glycation and AGE-modified mitochondria can induce cellular stress responses and autophagy via a RAGE-mediated mechanism. Modifying mitochondria with O-GlcNAcylation may prevent mitochondrial protein glycation and increase the viability of mitochondria when transferred into neurons.

### O-GlcNAcylated DJ1 counteracts glycation of mitochondrial protein

**Fig. 2:**
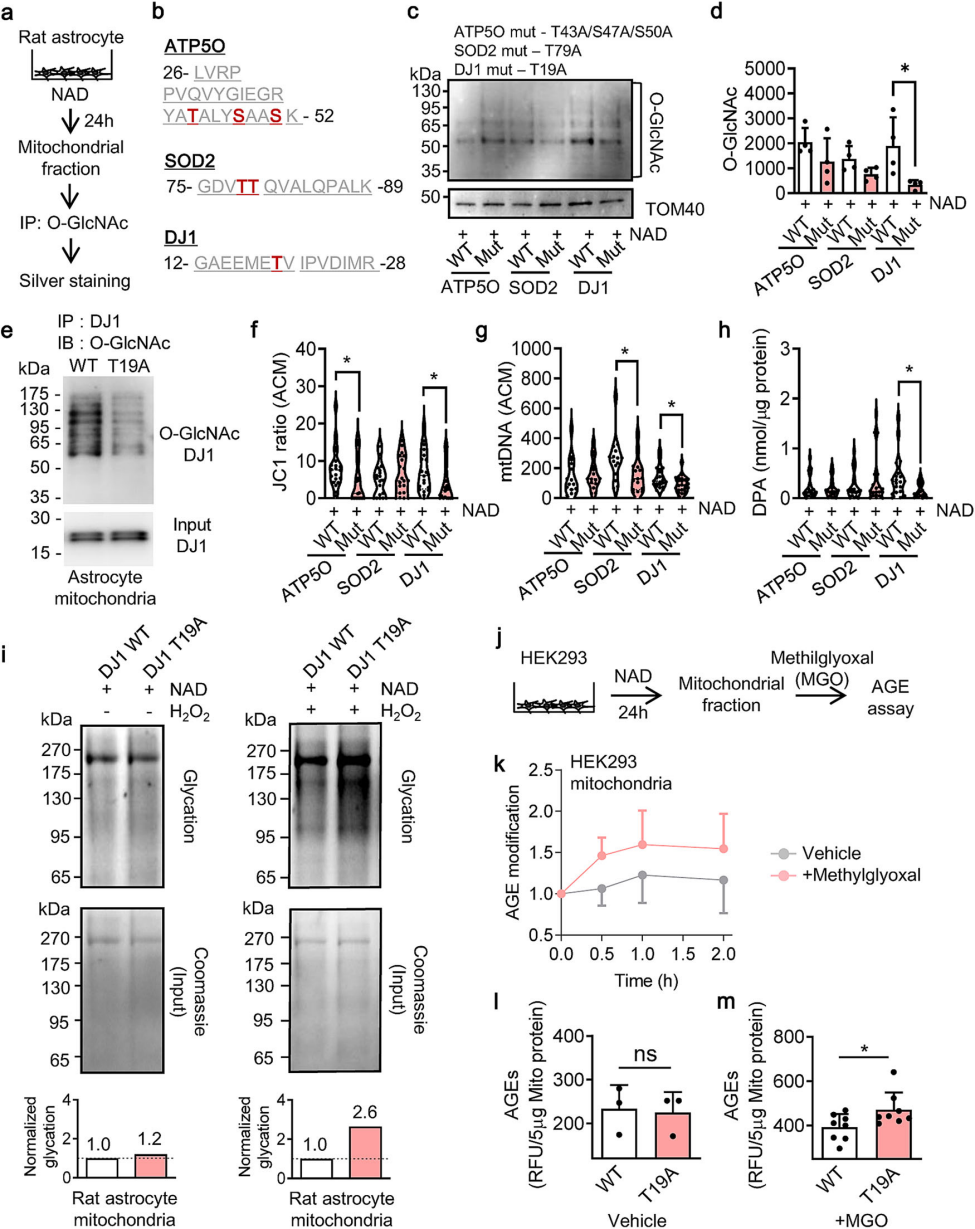
O-GlcNAcylated DJ1 counteracts glycation of mitochondrial protein. **a** Rat astrocytic CD38 signaling was stimulated by nicotinamide adenine dinucleotide (NAD, 5 mM) for 24 h. Following immunoprecipitation with O-GlcNAc antibody, silver staining was performed. Gels with target bands (rectangles) were cut out for Mass Spectrometry analysis. **b** Mass Spectrometry identified the potential mitochondrial protein candidates (ATP5O, SOD2, DJ1) for O-GlcNAcylation. IP: immunoprecipitation. **c** Based on identified serine/threonine resides by Mass Spectrometry, mutation (s) was introduced in ATP5O (T43A/S47A/S60A), SOD2 (T79A), and DJ1 (T19A). Representative image for western blot analysis. Note: Loading controls (TOM40) were run on separate membranes. **d** Quantification of western blots confirmed that mutation in SOD2 (T79A) or DJ1 (T19A) decreased O-GlcNAc-modified proteins following NAD treatment, but ATP5O mutation (T43A/S47A/S60A) showed less impact on total O-GlcNAcylation (ATO5O; *n* = 4, SOD2; *n* = 4, DJ1; *n* = 4). All values are mean ± SD. Unpaired *t* test (two-tails), *P=0.0359. **e** DJ1 O-GlcNAcylation was clearly decreased by mutation T19A in astrocytic mitochondria. Note: DJ1 O-GlcNAcylation was validated once by combining triplicated wells. Input was run on separate membrane. **f** Extracellular mitochondrial membrane potential (JC1) in astrocyte-conditioned media (ACM) (ATO5O; *n* = 20, SOD2; *n* = 20, DJ1/Park7; *n* = 20). All values are mean ± SD. ATP5O: Unpaired *t* test (two-tails), *P = 0.0417. DJ1: Unpaired *t* test (two-tails), *P = 0.0373. **g** mtDNA (ND-1) measurement in ACM (ATO5O; *n* = 15, SOD2; *n* = 15, DJ1/Park7; *n* = 23). All values are mean ± SD. SOD2: Unpaired *t* test (two-tails), *P = 0.0286. DJ1: Unpaired *t* test (two-tails), *P = 0.0116. **h** Dipicolinic acid (DPA) measurement in ACM (ATO5O; *n* = 20, SOD2; *n* = 20, DJ1/Park7; *n* = 20). All values are mean ± SD. DJ1: Unpaired *t* test (two-tails), *P = 0.0117. **i** DJ1 WT or T19A was introduced into rat astrocytes. Then NAD (5 mM) was treated for 24 h. Glycation assay revealed that DJ1 T19A mutation exacerbated mitochondrial protein glycation under oxidative stress condition with H_2_O_2_ (100 μM). Note: Mitochondrial glycation was validated once by combining triplicated wells. Input was detected on the same membrane. **j** Following induction of DJ1 WT or T19A, rat astrocytes or HEK293 cells were stimulated by NAD (5 mM), then mitochondria were isolated for further assay to evaluate resistance to protein glycation induced by methylglyoxial (50 μM). **k** Methylglyoxial (MGO, 50 μM) increased mitochondrial protein glycation after the 30-min exposure to mitochondria isolated from HEK293 (Vehicle; *n* = 3, MGO; *n* = 3). All values are mean ± SD. **l** Vehicle (PBS) treatment did not impact mitochondrial protein glycation in mitochondria isolated from HEK293 regardless of T19A (*n* = 3). All values are mean ± SD. **m** At 30 min post-stimulation with MGO, DJ1 mutation with T19A exacerbated mitochondrial protein glycation in mitochondria isolated from HEK293 (*n* = 11). All values are mean ± SD. Unpaired *t* test (two-tails), *P = 0.0375.

To further investigate this idea that O-GlcNAcylation in mitochondria can improve the efficacy and benefit of mitochondrial transfer, we asked what mitochondrial proteins were modified with O-GlcNAc. To identify O-GlcNAcylated proteins and the potential target sites of O-GlcNAcylation, rat astrocytes were stimulated with NAD to induce O-GlcNAcylation in mitochondria, then we isolated mitochondria and extracted O-GlcNAcylated proteins by immunoprecipitation using O-GlcNAc antibody followed by silver staining (Fig. 2a). Mass spectrometry of the gel samples revealed peptide sequences of ATP5O, SOD2, and DJ1 that included residues of serine/threonine relevant to O-GlcNAc targets (Fig. 2b). To assess causality, we attempted a loss-of-function experiment by introducing mutation(s) in serine or threonine of the three target proteins (ATP5O (T43A/S47A/S50A), SOD2 (T79A), DJ1 (T19A)). Western blots confirmed that mutations in DJ1 significantly decreased O-GlcNAcylation in mitochondrial proteins (Fig. 2c, d, Supplementary Fig. 1d). In contrast, ATP5O or SOD2 mutation had less impact on O-GlcNAcylation (Fig. 2c, d). To further assess DJ1 O-GlcNAcylation, we extracted DJ1 from astrocytes which were amplified by either DJ1 WT or DJ1 T19A. When DJ1 had mutation T19A, DJ1 O-GlcNAcylation was clearly decreased (Fig. 2e, Supplementary Fig. 1e**)**, suggesting that DJ1 may be a key target for O-GlcNAcylation. Strikingly, DJ1 mutation T19A significantly decreased mitochondrial functional parameters including mitochondrial membrane potential, mtDNA, and dipicolinic acid in ACM (Fig. 2f–h), suggesting that O-GlcNAcylated DJ1 may be the main supporter of extracellular mitochondrial function.

DJ1 is a cytoprotective pleiotrophic enzyme with glyoxalase and deglycase activities^18^. These enzyme activities may directly or indirectly contribute to prevent protein glycation (AGE-modification) and protect mitochondria^19^. Therefore, we assessed the role of O-GlcNAcylation on the ability of DJ1 to counteract mitochondrial protein glycation. Blocking O-GlcNAc-modification of DJ1 with mutation (T19A) exacerbated mitochondrial protein glycation after oxidative stress (Fig. 2i, Supplementary Fig. 1f). To further test this idea, we used methylglyoxal (MGO) to increase AGE in isolated mitochondria^20^ (Fig. 2j). As expected, in normal mitochondria, AGE levels increased over time after MGO exposure (Fig. 2k). In contrast, in mitochondria with the DJ1 mutation T19A, MGO-induced protein glycation was significantly worsened (Fig. 2l, m). Taken together, these findings suggest that O-GlcNAcylation in Thr19 of the mitochondrial protein DJ1 may play an important role to counteract glycation.

### O-GlcNAcylated mitochondria mediate pro-survival signals in human neurons

**Fig. 3:**
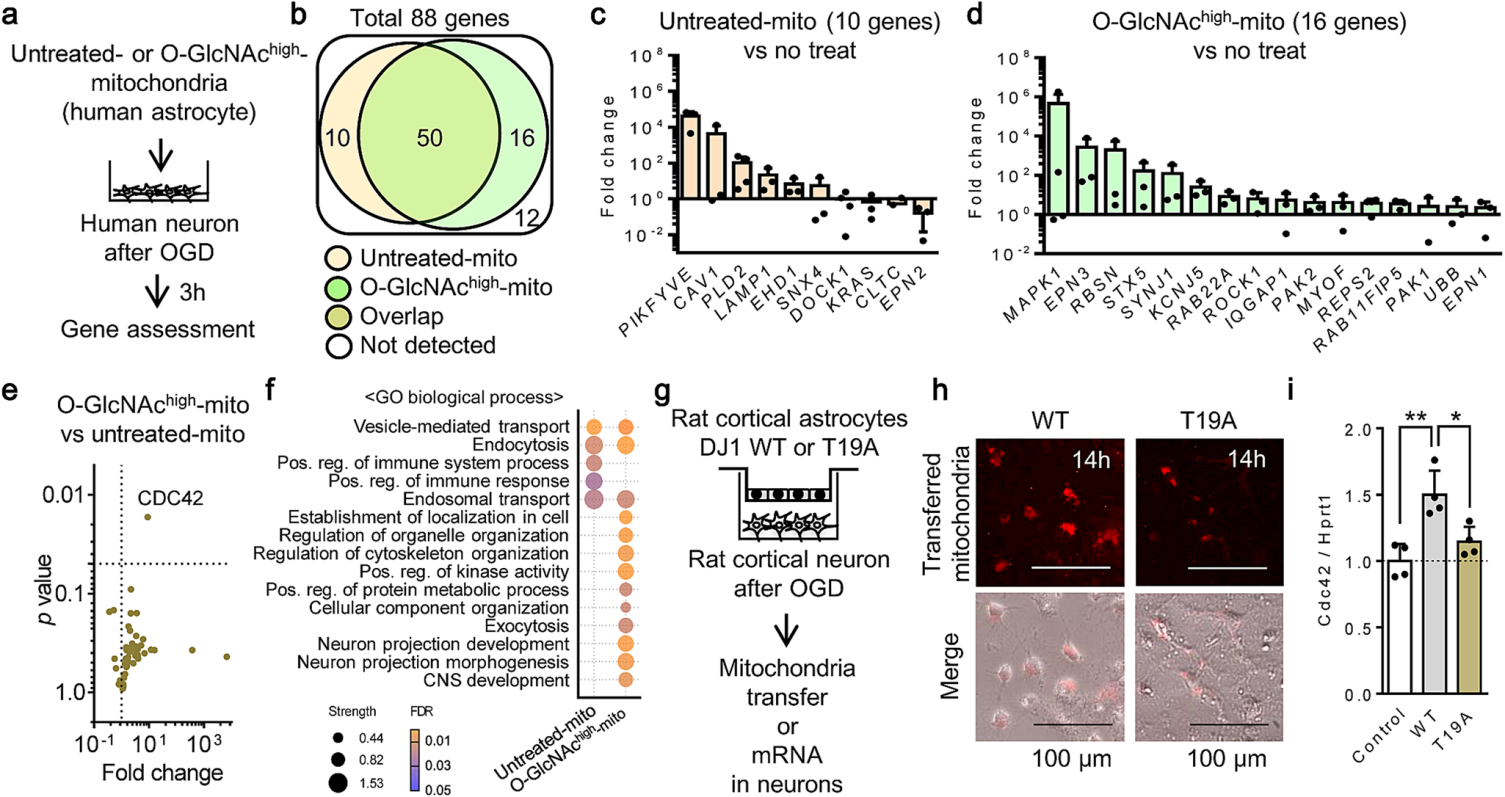
DJ1 O-GlcNAcylated mitochondria transfer activates intracellular regulatory pathways in human neurons. **a** Mitochondria isolated from human astrocytes were incubated with UDP-GlcNAc (32.5 µg) and O-GlcNAc transferase (OGT) (0.66 μg) for 30 min at 37 ^o^C. Then, O-GlcNAc-modified mitochondria (O-GlcNAc^high^-mitochondria, 2 µg/well) or unmodified control mitochondria susceptible to glycation (untreated mitochondria, 2 µg/well) were treated in human cortical neurons following oxygen-glucose deprivation (OGD) for 2 h. **b** Of total 88 gene sets, fifty genes were commonly expressed in two groups while 10 or 16 genes were uniquely detected in either group of untreated control mitochondria or O-GlcNAc^high^-mitochondria treatment, respectively. Twelve genes were undetected. **c** Unique genes in neurons treated with untreated control mitochondria (*n* = 3). All values are mean ± SD. **d** Unique genes in neurons treated with O-GlcNAc^high^-mitochondria (*n* = 3). All values are mean ± SD. **e** Volcano plot of genes that were commonly expressed in both groups. *CDC42* was significantly upregulated in neurons treated with O-GlcNAc^high^-mitochondria (*n* = 3). **f** Gene ontology (GO) enrichment (Biological process) showed that O-GlcNAc^high^-mitochondria treatment upregulated many cellular regulatory pathways including the regulation of organelle, cytoskeleton, and kinase activity. **g** A transwell-utilized coculture between astrocytes and neurons to evaluate the effect of mitochondria transfer on *CDC42* mRNA in the recipient neurons. **h** Stimulated astrocytes with NAD (5 mM) transferred mitochondria to 2 h OGD-neurons cultured in the bottom cells at 14 h. Mitochondria from DJ1 WT astrocytes remained in the recipient neurons more than ones from DJ1 T19A astrocytes. **i** *CDC42* was upregulated in the recipient neurons at 24 h after the coculture with DJ1 WT astrocytes but not with T19A astrocytes (*n* = 4). All values are mean ± SD. One-way ANOVA: *F*(2,9) = 12.86, *P* = 0.0023 followed by Tukey test, *P < 0.05, **P < 0.01.

Thus far, we have demonstrated the utility of mitochondrial O-GlcNAcylation in rodent cells. Here, we asked whether these beneficial signals can also be detected in human cells. Mitochondria were collected from human astrocytes and then added to human neurons that were previously damaged with oxygen-glucose deprivation. Then RNA was isolated from the neurons, and gene expression profiles were examined with an endocytosis gene array, comparing responses in neurons treated with untreated control mitochondria or O-GlcNAc^high^-mitochondria. (Fig. 3a). Of total 88 gene sets, fifty genes were commonly expressed in the two groups while 10 or 16 genes were uniquely detected in either group of control mitochondria or O-GlcNAc^high^-mitochondria, respectively (Fig. 3b). Another 12 genes were undetectable in both groups (Fig. 3b). Importantly, the gene array implicated that *PIKFYVE* and *LAMP1* were unique genes upregulated in neurons when control mitochondria were treated (Fig. 3c), confirming the upregulation of lysosomal degradation activity. On the other hand, O-GlcNAc^high^-mitochondria uniquely increased pro-survival regulators such as *MAPK1*, *PAK1, PAK2* (Fig. 3d) along with upregulation of *CDC42* that is highly associated with these kinase activities and also prevents lysosome-mediated degradation machinery^21^ (Fig. 3e). Gene ontology (GO) enrichment analysis showed that control mitochondria -treated neurons showed gene upregulation in immune response (Fig. 3f) while O-GlcNAc^high^-mitochondria-treated neurons upregulated many cellular regulatory processes including the regulation of organelle, cytoskeleton, kinase activity, and neuron projection development/morphogenesis (Fig. 3f). To show that these outcomes were specifically dependent on O-GlcNAc-modified Thr 19 of DJ1, DJ1 WT or T19A mutant astrocytes were co-cultured with neurons in a transwell system (Fig. 3g). As expected, after 14 h of coculture, mitochondria were transferred into neurons in this system. Mitochondria from DJ1 WT astrocytes were retained in the recipient neurons at a higher level compared to mitochondria from DJ1 T19A astrocytes (Fig. 3h). Importantly, neuronal *CDC42* was significantly upregulated when mitochondria were transferred from WT DJ1-amplified astrocytes (Fig. 3i). Collectively, these data suggest that without O-GlcNAcylation, transplantation of mitochondria may induce a pathogen-clearance response in human neurons, whereas mitochondria with O-GlcNAc-DJ1 may escape the lysosomal degradation pathway and increase beneficial pro-survival signals.

### O-GlcNAcylation promotes neuroprotection and recovery after stroke

**Fig. 4:**
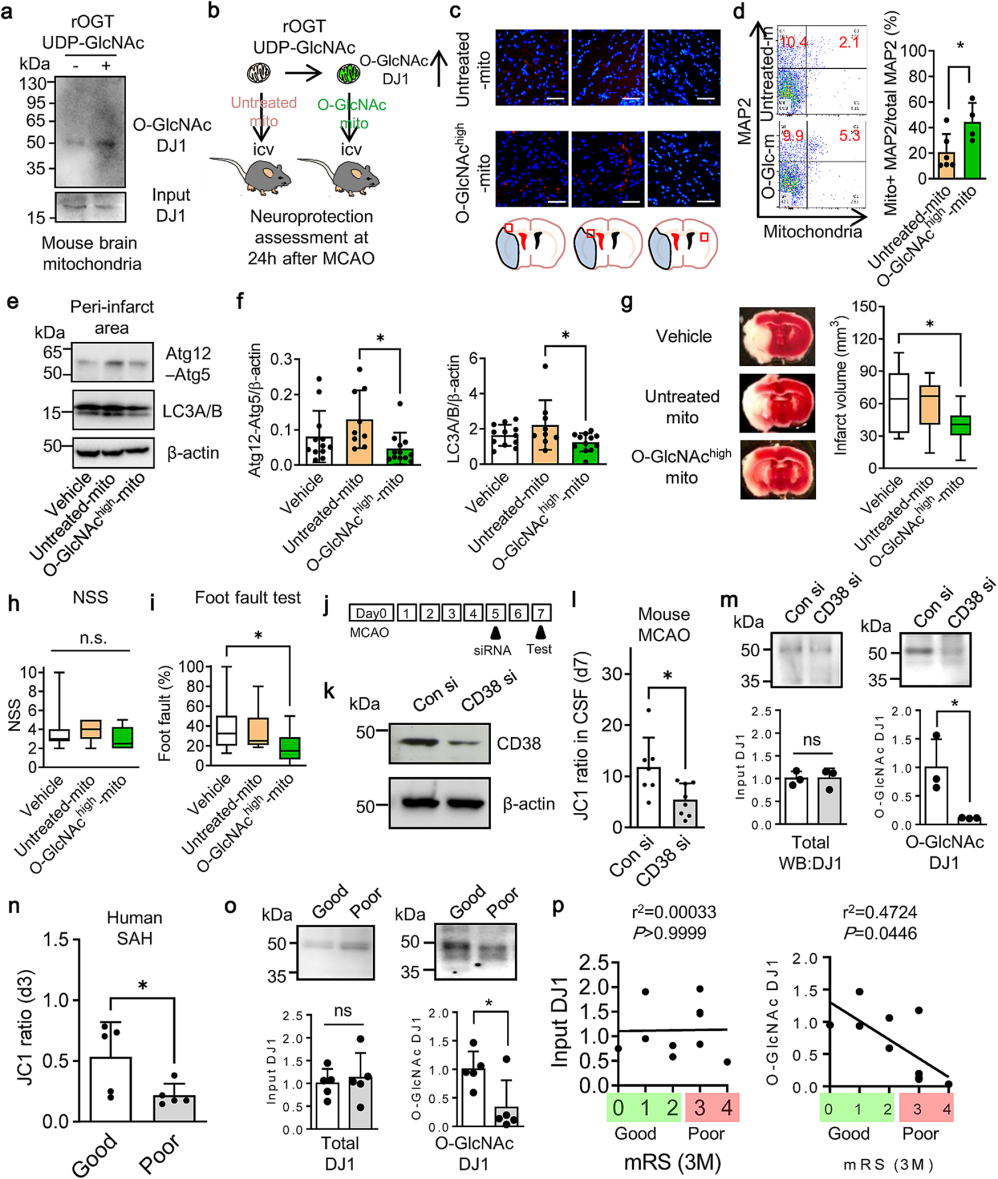
DJ1 O-GlcNAcylation promotes neuroprotection and recovery after stroke. **a** Mitochondria isolated from intact cerebral cortex in male C57BL/6J mice were incubated with UDP-GlcNAc (32.5 µg) and O-GlcNAc transferase (OGT) (0.66 μg) for 30 min at 37 ^o^C. This process amplified DJ1 O-GlcNAcylation in isolated mitochondria. Note: DJ1 O-GlcNAcylation was initially validated once after immunoprecipitation with DJ1 antibody, then we treated untreated or modified mitochondria in a mouse model of focal ischemia thereafter. Input was run on separate membrane. **b** Male C57BL/6J mice were subjected to transient 60 min focal ischemia (middle cerebral artery occlusion: MCAO), and untreated control mitochondria (6 μg/5 μL) or O-GlcNAc^high^-mitochondria (6 μg/5 μL) were injected into lateral ventricles after reperfusion. Infused O-GlcNAcylated mitochondria were found in the ipsilateral hemisphere but not contralateral hemisphere. **c** Immunohistochemistry demonstrated that infused O-GlcNAc^high^-mitochondria were more found in the ipsilateral hemisphere compared to untreated control mitochondria. Scale: 100 μm. **d** FACS analysis showed that O-GlcNAc^high^-mitochondria were more remained in MAP2+ neurons compared to untreated control mitochondria (Cont-mito *n* = 6, O-GlcNAc-mito *n* = 4). All values are mean ± SD. Unpaired *t* test (two-tails), *P = 0.0349. **e** Western blot confirmed that autophagic responses (Atg12-5 complex, LC3A/B) were lower in O-GlcNAc^high^-mitochondria infusion compared to untreated control mitochondria in the peri-infarct region at 24 h post-stroke. Note: β-actin signals were obtained by reuse of the membranes after stripping Atg12-Atg5 signals. **f** Quantification of western blots (vehicle *n* = 12, Cont-mito *n* = 9, O-GlcNAc-mito *n* = 12). All values are mean ± SD. Atg12-Atg5: One-way ANOVA: *F*(2,30) = 3.907, *P* = 0.0310 followed by Tukey test, *P < 0.05. LC3A/B: One-way ANOVA: *F*(2,30) = 3.273, *P* = 0.0518 followed by Tukey test, *P < 0.05. **g** Treatment with O-GlcNAc^high^-mitochondria induced better protective effect than untreated control mitochondria (Vehicle *n* = 15, Untreated-mito *n* = 11, O-GlcNAc-mito *n* = 14). All values are mean ± SD. One-way ANOVA: *F*(2,37) = 4.527, *P* = 0.0174 followed by Tukey test, *P<0.05. **h** NSS score after treatment with O-GlcNAc^high^-mitochondria or untreated control mitochondria (Vehicle *n* = 14, Untreated-mito *n* = 11, O-GlcNAc-mito *n* = 14). All values are mean ± SD. **i** Treatment with O-GlcNAc^high^-mitochondria significantly improved foot fault test than untreated control mitochondria (Vehicle *n* = 14, Untreated-mito *n*= 11, O-GlcNAc-mito n = 14). All values are mean ± SD. One-way ANOVA: *F*(2,36) = 4.147, *P* = 0.0240 followed by Tukey test, *P < 0.05. **j** Male C57BL/6J mice were subjected to transient 60 min focal ischemia and control siRNA or CD38 siRNA was injected into lateral ventricles at 5 days after stroke, consistent with our previous report^22^. The transfection efficacy was validated once in this study. **k** Western blot analysis demonstrated siRNA suppression of CD38 at day 7 post-stroke in mice. Note: Loading control (β-actin) was run on separate membrane. **l** CSF samples were collected at 7 days post-stroke. Extracellular mitochondrial JC1 analysis demonstrated that CD38 siRNA significantly decreased JC1 values (Conn si; *n* = 7, CD38 si; *n* = 8). All values are mean ± SD. Unpaired *t* test (two-tails), *P = 0.0186. **m** Immunoprecipitation with DJ1 antibody followed by western blot with O-GlcNAc antibody confirmed that CD38 siRNA decreased DJ1 O-GlcNAcylation while there was no significant difference in total DJ1 (*n* = 3). All values are mean ± SD. Unpaired *t* test (two-tails), *P = 0.0351. Note: Input and IP products were run on separate membrane. **n** Human CSF samples were collected from subarachnoid hemorrhagic stroke patients (*n* = 10). In these cohorts, higher mitochondrial membrane potentials in the CSF were correlated with good clinical recovery at 3 months after SAH onset (mRS: 0–2 - good, *n* = 5, mRS: 3–4 - poor, *n* = 5). All values are mean ± SD. Unpaired *t* test (two-tails), *P = 0.0463. **o** Assessment of O-GlcNAcylation in DJ1 in human CSF samples collected from subarachnoid hemorrhagic stroke patients (*n* = 10). We analyzed patients with good (*n* = 5) or poor (*n* = 5) clinical recovery at 3 months after SAH onset. O-GlcNAc-modification in DJ1 was higher in patients with good clinical recovery at 3 months after SAH (mRS: 0–2 - good, *n* = 5, mRS: 3–4 - poor, *n* = 5). There was no significant difference in total protein expressions. All values are mean ± SD. Unpaired *t* test (two-tails), *P = 0.0306. Note: Input and IP products were run on separate membrane. **p** Although there was no association between total DJ1 protein expression and outcome (*r*^2 ^= 0.00033, *P* > 0.9999), the overall level of O-GlcNAcylated proteins appeared to be positively correlated with good clinical recovery (*r*^2 ^= 0.4724, *P* = 0.0446).

Finally, we asked whether mitochondria modified with O-GlcNAc are indeed beneficial in vivo. Mitochondria were isolated from mouse cerebral cortex and then treated with UDP-GlcNAc and OGT in order to amplify O-GlcNAcylation (Fig. 4a, Supplementary Fig. 1g). Then, O-GlcNAc^high^- mitochondria or untreated control mitochondria were allografted into the ventricles of mice subjected to 60 min of focal cerebral ischemia (Fig. 4b). Consistent with our in vitro results, O-GlcNAc^high^- mitochondria showed higher retention levels in neurons (Fig. 4c, d). Unmodified mitochondria increased autophagy markers (Atg12-Atg5 complex and LC3A/B) while O-GlcNAc-mitochondria did not increase these markers in ipsilateral cortex (Fig. 4e, f, Supplementary Fig. 1h). Most importantly, O-GlcNAcylation improved the allograft-mediated neuroprotective effect after focal cerebral ischemia, i.e., transplantation of modified mitochondria resulted in significantly smaller infarct volumes in accompanied by better functional outcomes compared to unmodified mitochondria (Fig. 4g–i).

As part of an endogenous protective response, CD38 signaling mediates the transfer of mitochondria from astrocytes to vulnerable neurons after cerebral ischemia^22^. If O-GlcNAcylation of DJ1 is indeed important for extracellular mitochondrial function, then one should expect that interfering with this CD38 process should correlate with a decrease in O-GlcNAcylated DJ1. Once again, mice were subjected to 60 min focal cerebral ischemia, and 5 days later, intracerebroventricular infusion of control siRNA or CD38 siRNA was performed (Fig. 4j, k, Supplementary Fig. 1i). CD38 suppression with siRNA significantly decreased JC1 membrane potential of extracellular mitochondria in cerebrospinal fluid (CSF) (Fig. 4l) along with reducing O-GlcNAcylation of DJ1 without changing total DJ1 levels (Fig. 4m, Supplementary Fig. 1j).

**Fig. 5:**
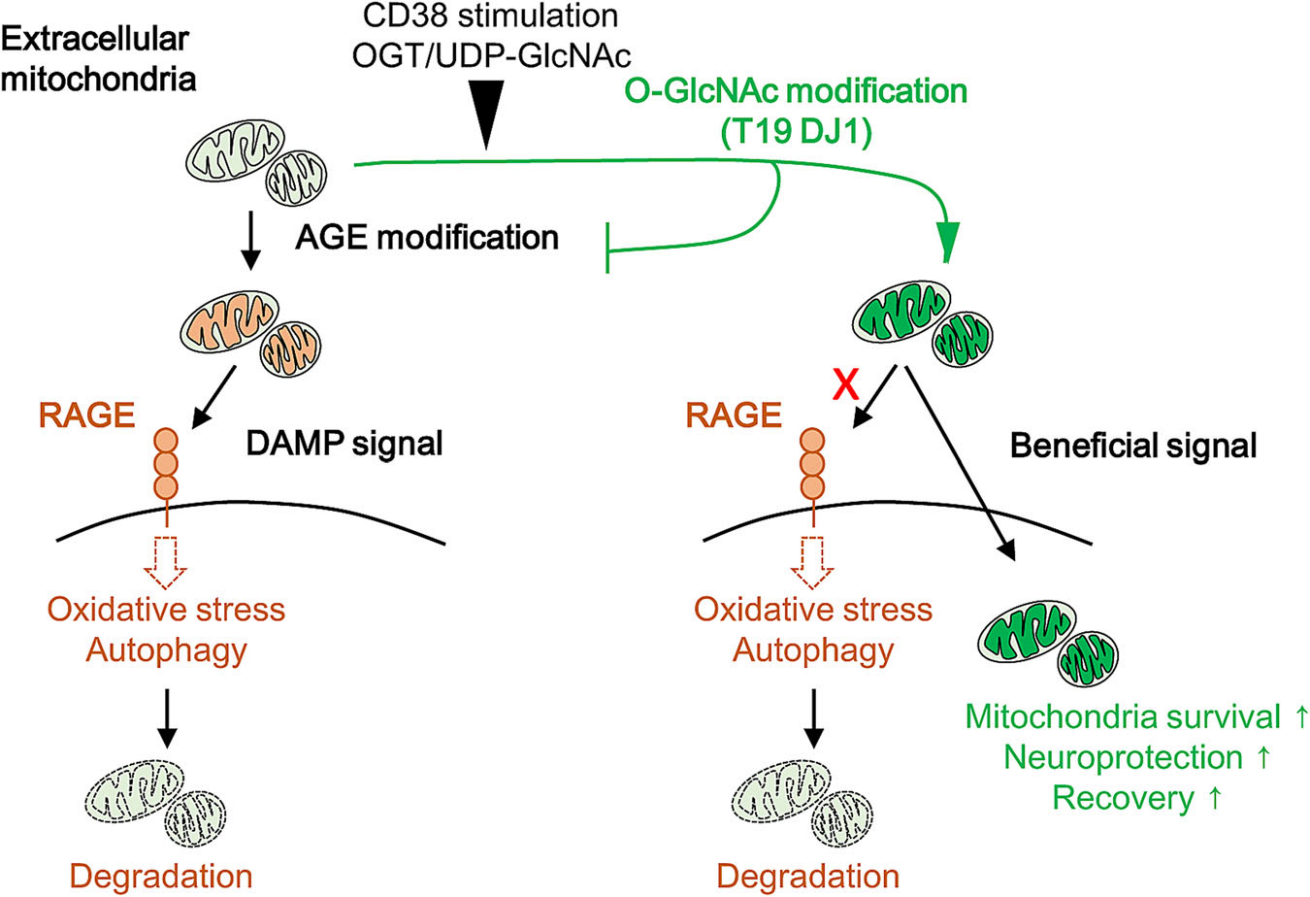
Mitochondrial O-GlcNAcylation is regulated by CD38 or OGT/UDP-GlcNAc and O-GlcNAc-modified mitochondria support neuronal survival and plasticity after brain ischemia and injury. Without O-GlcNAcylation, mitochondria become susceptible to glycation and induce RAGE-mediated stress response in the recipient neuron.

To explore translational potential, we turned to CSF collected from patients with subarachnoid hemorrhagic stroke (SAH). Consistent with previous observations^23^, higher mitochondrial membrane potentials in CSF extracellular mitochondria correlated with good clinical recovery at 3 months after SAH (Fig. 4n). Although there was no association between total DJ1 protein expression and outcome, the overall level of O-GlcNAcylated proteins appeared to be positively correlated with good clinical recovery (Fig. 4o, p, Supplementary Fig. 1k). Collectively, these human and mouse data suggest that the level of DJ1 O-GlcNAcylation in extracellular mitochondria may contribute to endogenous protective responses as well as improve the efficacy of exogenous allografts in stroke (Fig. 5).

## Discussion

More than 400 completed and ongoing clinical trials for mitochondrial-targeted medical intervention have been registered at ClinicalTrials.gov. However, medicines targeting mitochondria remain to be fully translated into clinical efficacy. Our present study demonstrates that normal mitochondria may be susceptible to glycation and produce danger signals while O-GlcNAc-modification of DJ1 may increase mitochondrial resistance to glycation, thus resulting in beneficial mitochondria transplantation in the CNS.

Glycation and glycosylation are two distinct processes regulated by non-enzymatic and enzymatic reactions, respectively. AGE-adducts act as DAMPs and are known to act as deleterious signals that increase in aging, inflammation, and metabolic and vascular diseases^24^. On the other hand, O-GlcNAcylation may be a beneficial signal that is diminished in aged brains compared to young brains after cerebral ischemia^25^. Moreover, pharmacologic amplification of O-GlcNAcylation may improve neuroprotection, inhibit pro-inflammatory signaling in microglia and promote stroke recovery^26,27^. In this study, data from rodent and human models showed that isolated normal mitochondria were susceptible to AGE-modification and induced RAGE-mediated damaging signals in neurons after mitochondrial transfer, whereas DJ1 O-GlcNAcylation was crucial for sustaining extracellular mitochondrial functionality as well as promoting neuroprotection and recovery after cerebral ischemic injury. Altogether, levels of DJ1 O-GlcNAcylation may serve as potential biomarkers for recovery from CNS injury and disease.

DJ1 is encoded by the human PARK7 gene, and is found in both outer membrane and inner compartments of mitochondria^28^. As a multifunctional protein that regulates ATP production and mitochondrial permeability transition pore opening, DJ1 serves as an oxidative stress response protein with anti-glycation and anti-aging abilities^29^. Cys106, His126, and Glu18 of DJ1 may comprise a critical active site pocket to recognize large substrates by binding the glycated portion^30^. Given that DJ1 has both glyoxalase and deglycase activities, the localization of DJ1 in extracellular mitochondria may play a critical role to increase resistance to glycation in pathological environments. Our current study revealed an unappriciated role of O-GlcNAcylation site Thr19 of DJ1 in supporting ability to counteract mitochondrial protein glycation under stress conditions such as oxidative stress and methylglyoxal that are commonly involved in various pathologies^20^.

Nevertheless, there are a few caveats that warrant further investigation. First, how the treatment with OGT and UDP-GlcNAc amplifies O-GlcNAcylation in isolated mitochondrial proteins remains to be fully investigated. DJ1 is found in outer membrane. Therefore, even a large molecule such as OGT may be able to catalyze attachment of the O-GlcNAc if serine/threonine residues are exposed. To ensure the accessibility of OGT, molecular structure of DJ1 on the location of outer membrane of mitochondria should be investigated. Moreover, we are fully aware of that DJ1 is not only the protein modified with O-GlcNAc in mitochondria. It has been shown that O-GlcNAcylated VDAC may have roles to attenuate oxidative stress and inhibit the formation of mitochondrial permeability transition pore induced by calcium overload^31^. How is the mitochondrial proteome modified and how other O-GlcNAcylated mitochondrial proteins may protect extracellular mitochondria should be systematically explored in future studies. Second, we evaluated only O-GlcNAcylation in this study. But we are aware of the importance of other post-translational modification such as phosphorylation, acetylation, and ubiquitination. Future studies should investigate how the dynamic balance of various protein modification events affects the viability of extracellular mitochondria and the efficacy of cellular transfer and protection. Third, human CSF samples collected from SAH patients were analyzed in this study. Although our results demonstrated that levels of DJ1 O-GlcNAcyation in CSF was correlated with good clinical recovery at 3 month after SAH onset, we acknowledge that the impact of O-GlcNAc-modification in DJ1 in ischemic stroke in human remains unclear. Lastly, how DJ1 O-GlcNAc-modification of mitochondria is affected in broader pathophysiological conditions remains to be fully elucidated. O-GlcNAc post-translational modification may be attenuated during inflammation and aging in astrocytes^32^. Therefore, whether manipulating endogenous O-GlcNAcylation can continue to work in the inflamed and/or aging CNS needs to be further addressed.

Mitochondrial integrity is critical for CNS recovery after injury or disease^33,34^. Our study suggests that extracellular mitochondria may be susceptible to AGE-modification that induces unfavorable responses in recipient neurons after transfer. O-GlcNAcylation of mitochondrial DJ1 may increase extracellular mitochondria resistance to glycation and improve the neuroprotective capacity of mitochondria for therapies in stroke and other CNS diseases.

### Supplementary information

Supplementary Information

Description of Additional Supplementary Files

Supplementary Data 1

Reporting Summary

## Data Availability

Uncropped western blot replicates for quantified figures are presented in Supplementary Fig. 1. The source data for all the graphs presented in figures are available in Supplementary Data 1. The mass spectrometry proteomics data have been deposited to the ProteomeXchange Consortium via the PRIDE partner repository with the dataset identifier PXD045316 and 10.6019/PXD045316. Other data generated during this study are available from the corresponding author upon the appropriate request to khayakawa1@mgh.harvard.edu.
