# Supplementary material for: O-GlcNAcylation is essential for therapeutic mitochondrial transplantation
Source: Commun Med (Lond). 2023 Nov 25;3:169. doi: 10.1038/s43856-023-00402-w (PMC10676354; doi:10.1038/s43856-023-00402-w)

Supplementary Figure 1: Uncropped or replicate raw blots

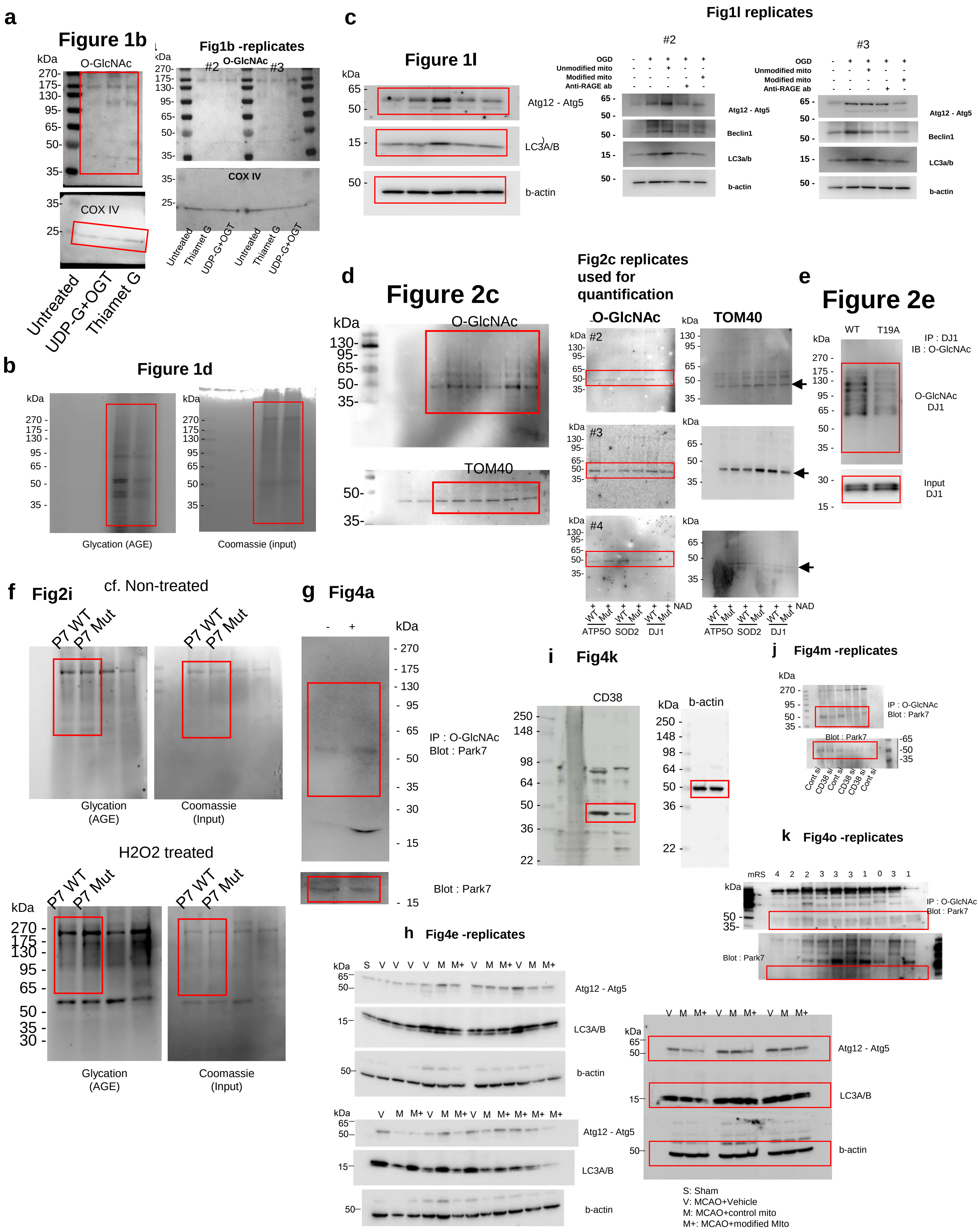

Supplement: Supplementary file 1 — Supplementary Information [file 43856_2023_402_MOESM1_ESM.pdf]
