# Supplementary material for: O-GlcNAcylation is essential for therapeutic mitochondrial transplantation
Source: Commun Med (Lond). 2023 Nov 25;3:169. doi: 10.1038/s43856-023-00402-w (PMC10676354; doi:10.1038/s43856-023-00402-w)
Supplement: Supplementary file 2 — Description of Additional Supplementary Files [file 43856_2023_402_MOESM2_ESM.pdf]

Description of Additional Supplementary Files

File Name: Supplementary Data 1
Description: All values (biological replicates and technical replicates) in figures 1, 2, 3, and are demonstrated.
